# MicroRNAs Differentially Expressed in Actinic Keratosis and Healthy Skin Scrapings

**DOI:** 10.3390/biomedicines11061719

**Published:** 2023-06-15

**Authors:** Maria Vincenza Chiantore, Marco Iuliano, Roberta Maria Mongiovì, Fabiola Luzi, Giorgio Mangino, Lorenzo Grimaldi, Luisa Accardi, Gianna Fiorucci, Giovanna Romeo, Paola Di Bonito

**Affiliations:** 1 EVOR Unit, Department of Infectious Diseases, Istituto Superiore di Sanità, 00161 Rome, Italy; luisa.accardi@iss.it (L.A.); gianna.fiorucci@cnr.it (G.F.); paola.dibonito@iss.it (P.D.B.); 2 Department of Medico-Surgical Sciences and Biotechnologies, Sapienza University of Rome—Polo Pontino, 04100 Latina, Italy; marco.iuliano@uniroma1.it (M.I.); robertamaria.mongiovi@gmail.com (R.M.M.); giorgio.mangino@uniroma1.it (G.M.); lorenzo.grimaldi@uniroma1.it (L.G.); giovanna.romeo@uniroma1.it (G.R.); 3 Plastic and Reconstructive Surgery, San Gallicano Dermatologic Institute IRCCS, 00144 Rome, Italy; fabiolaluzi@yahoo.it; 4 Institute of Molecular Biology and Pathology, CNR, 00185 Rome, Italy

**Keywords:** actinic keratosis, cSCC, microRNA, biomarkers

## Abstract

Actinic keratosis (AK) is a carcinoma in situ precursor of cutaneous squamous cell carcinoma (cSCC), the second most common cancer affecting the Caucasian population. AK is frequently present in the sun-exposed skin of the elderly population, UV radiation being the main cause of this cancer, and other risk factors contributing to AK incidence. The dysregulation of microRNAs (miRNAs) observed in different cancers leads to an improper expression of miRNA targets involved in several cellular pathways. The TaqMan Array Human MicroRNA Card assay for miRNA expression profiling was performed in pooled AK compared to healthy skin scraping samples from the same patients. Forty-three miRNAs were modulated in the AK samples. The expression of miR-19b (*p* < 0.05), -31, -34a (*p* < 0.001), -126, -146a (*p* < 0.01), -193b, and -222 (*p* < 0.05) was validated by RT-qPCR. The MirPath tool was used for MiRNA target prediction and enriched pathways. The top DIANA-mirPath pathways regulated by the targets of the 43 miRNAs are TGF-beta signaling, Proteoglycans in cancer, Pathways in cancer, and Adherens junction (7.30 × 10^−10^ < *p* < 1.84 × 10^−8^). Selected genes regulating the KEGG pathways, i.e., *TP53*, *MDM2*, *CDKN1A*, *CDK6*, and *CCND1*, were analyzed. MiRNAs modulated in AK regulate different pathways involved in tumorigenesis, indicating miRNA regulation as a critical step in keratinocyte cancer.

## 1. Introduction

Actinic keratosis (AK) is currently recognized as a carcinoma in situ precursor of cutaneous squamous cell carcinoma (cSCC) [1], the second most common keratinocyte cancer affecting the White population worldwide, and its incidence has increased with the population aging [1,2,3]. Several risk factors influence this neoplasia of keratinocytes, such as genetic factors, family history for cSCC, immune system status, prolonged sun exposure, artificial UV tanning, male sex, fair skin, radiotherapy, and exposure to specific chemicals [4]. Recently, specific beta human papillomaviruses (HPV) have emerged as cofactors in the development of AK and cSCC [5], but cumulative UV exposure is the main risk factor for cSCC. In addition, immunosuppression (e.g., transplant recipients and patients with hematologic cancers such as chronic lymphocytic leukemia) is associated with a higher incidence and more aggressive course of cSCC. AK frequently occurs in older individuals; it can spontaneously regress or evolve into cSCC [1]. The epidemiology of AKs reflects their causation by cumulative sun exposure, with the highest prevalence seen in pale-skinned people living in low latitudes and in the most sun-exposed body sites, namely the hands, forearms, and face.

In recent years, significant progress has been made in the detection of specific biomarkers for accurate cancer diagnosis and prognosis in cancer, and to guide personalized therapy by estimating outcome risks. In this context, microRNAs (miRNAs) have been shown to be attractive candidates as biomarkers in cancer pathophysiology and diagnosis [6]. The role of miRNAs in controlling the activity of cutaneous stem cells and in driving skin development and regeneration has been demonstrated. Some miRNAs are involved in controlling the expression of key genes of stem cell activity in healthy skin and hair follicles; therefore, the research of cSCC biomarkers has focused on these miRNAs [7]. The majority of cSCC arise from AK: it has been reported that certain miRNAs are differentially expressed in AK and in cSCC [7,8,9] and that this difference could be correlated with the progression from AK to invasive cancer [10].

In this study, the miRNA profiles of pooled AK scraping samples were compared to those obtained with pooled HS skin scraping samples from the same patients. Among these miRNAs, the expression of miR-19b, -31, -34a, -126, -146a, -193b, and -222 was validated. The analysis of the pathways regulated by miRNA target genes was performed by the DIANA tool mirPath v.3 with all the miRNAs modulated in AK samples. In addition, the expression of selected miRNA target genes, i.e., *TP53*, *MDM2*, *CDKN1A*, *CDK6*, and *CCND1*, was analyzed.

## 2. Materials and Methods

### 2.1. Clinical Samples Collection

The collection of clinical samples for this study was carried out in conjunction with our previously published studies, approved by the Ethical Committees of NIHMP (2014) and the San Gallicano Dermatological Institute (CE943/17; RS/1090/18) [11,12]. The investigations were carried out following the rules of the Declaration of Helsinki of 1975 (https://www.wma.net/what-we-do/medical-ethics/declaration-of-helsinki/, accessed on 14 January 2019), revised in 2013. Samples were from 20 patients (median age of 72) with a single lesion eligible for laser surgery. The AK samples were collected by scraping the lesions with a sterile spatula without reaching the dermis. The HS samples were collected with a second sterile spatula from the glabellar area, a source of thick skin, of each patient. Both AK and HS samples were immediately stored at −80 °C until processing.

### 2.2. MicroRNA Extraction and TaqMan Array Human MicroRNA A Card Analysis

Scraping skin samples were pooled (two different pools of 10 samples for each experimental group) and lysed by the mirVana miRNA detection kit (Applied Biosystems, Waltham, MA, USA), following the manufacturer’s procedure. The extracted RNA was retro-transcribed by using the TaqMan Micro-RNA Reverse Transcription Kit, and the Megaplex RT Primers and TaqMan Array Human MicroRNA A Card (Applied Biosystems, Waltham, MA, USA) was used to analyze the expression of multiple miRNA sequences in AK compared to HS samples. The obtained data were analyzed by using qPCR on Thermo Fisher Cloud (Thermo Fisher Scientific, Waltham, MA, USA) by setting global normalization, 35 as the maximum threshold cycle (Ct) and HS as reference.

### 2.3. Real Time RT-PCR

The results obtained by miRNA profiling were individually validated using specific TaqMan Small RNA assays following the manufacturer’s instructions (Applied Biosystems, Waltham, MA, USA). Data were normalized using U6 as endogenous control and expressed using the 2^−ΔΔCT^ method [13].

A total of 1 μg of extracted RNA of the AK and HS samples was retro-transcribed using the High-Capacity cDNA Reverse Transcription Kit (Applied Biosystem, Waltham, MA, USA), and cDNA products were analyzed by real-time RT-PCR using the SensiMix SYBR Hi-ROX Kit (Meridian Bioscience, Cincinnati, OH, USA). Data were normalized using as endogenous control HPRT-1 and expressed using the 2−ΔΔ*C*T method. Primers used are reported (Table 1).

### 2.4. Principal Component Analysis (PCA)

PCA was performed using the ClustVis website and the “PCA method” of the R package (https://biit.cs.ut.ee/clustvis/#editions, accessed on 9 February 2023) [19]. Shortly, the analysis was conducted on ΔCt of all the miRNAs that were eligible for selection from the TaqMan Array Human MicroRNA A Cards analysis of AK samples. To allow for a comparison, the ΔCt from the analysis on miRNAs of HS samples were used.

### 2.5. MiRNA Target Prediction and Enriched Pathways

The mirPath tool (version 3.0) was used to predict target genes of the differentially regulated miRNAs using the microT-CDS v.5.0 database and to retrieve KEGG (i.e., Kyoto Encyclopedia of Genes and Genomes) molecular pathways, considering *p* values lower than 0.05 as significant for pathway enrichment.

### 2.6. Statistical Analysis

Two-tailed *p* value Mann-Whitney *t* test was performed to compare two sample groups. *: *p* < 0.05; **: *p* < 0.01; ***: *p* < 0.001. The presented data are mean ± standard deviation (SD).

## 3. Results

### 3.1. MicroRNA Profiling of AK Samples and Matched Healthy Skin

The skin scrapings were collected from patients with one AK lesion at an early stage. As only a small amount of RNA could be extracted from these samples, miRNA profiling was performed with a pool of scraping extracts. Real-time PCR array analysis of 384 miRNAs, mainly expressed in the human genome, was performed with two different pools of 10 samples collected from AK and HS, as described in Materials and Methods.

**Figure 1:**
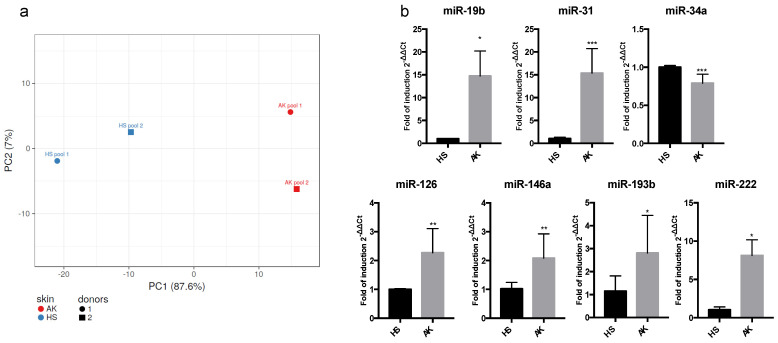
(**a**) Principal component analysis performed on ΔCts derived from the analysis of two couples of different pools of skin scrapings from AK and HS. Each dot represents the variance of the miRNA dataset for each sample type. PCA was performed by using ClustVis (https://biit.cs.ut.ee/clustvis/, accessed on 9 February 2023) algorithm. (**b**) MiRNA expression analysis performed in AK compared to HS. Total RNA from each sample was purified as described in Materials and Methods. Results were expressed as fold of induction or decrease using the 2^−ΔΔCT^ method [13] using RNU6 as a calibrator and HS as a control. The reported values represent the mean of three independent experiments ± the SD. *p*-values (*p*) are indicated with asterisks. *** *p* < 0.001; ** *p* < 0.01, * *p* < 0.05.

The principal component analysis (PCA) of the miRNA profiles was performed using the ClustVis website on ΔCt of all the miRNAs that were eligible for selection (Figure 1a). The miRNA dataset for each pool of samples was analyzed, and its variability was reduced and represented as a single dot on a first principal component PC1 vs. PC2 scatterplot. The PCA shows that the miRNA profiles of the two AK sample pools are different from the profiles of the two HS sample pools from the same donors. The PC1, which describes the majority of the variance, indicated that the miRNA datasets from the two AK pools were similar to each other but different from the miRNA datasets obtained from the HS pools. This suggested that the number of miRNAs with an altered expression in AK compared to HS was sufficient to allow for discrimination in PCA.

The miRNA profiles of AK and HS pools were analyzed using the Thermofisher Cloud to determine the differentially expressed miRNAs. Table 2 shows the complete list of miRNAs dysregulated in AK compared to the HS samples and the relative quantification.

Among the miRNAs differentially expressed in the AK samples, we selected seven miRNAs, i.e., miR-19b, -31, -34a, -126, -146a, -193b, and -222, based on their higher modulation with respect to the HS samples. Their altered expression was validated by specific TaqMan Small RNA assays. This analysis confirmed the results obtained by TaqMan Array Human MicroRNA A Card, showing that all these miRNAs were significantly upregulated, except for miR-34a, that was down-regulated in AK compared to HS (Figure 1b).

### 3.2. Pathways Regulated by Target Genes of miRNAs Modulated in AK Samples

In order to predict miRNA target and enriched pathways, the analysis with the DIANA tool mirPath v.3 was performed with all the 43 AK dysregulated miRNAs detected by the array. A total of 66 pathways were found to be regulated by target genes of these miRNAs, as reported in Table 3. Pathways are listed according to increasing *p*-value.

The first two pathways regulated by target genes of the selected miRNAs are TGF-beta signaling and Proteoglycans in cancer, followed by Pathways in cancer and Adherens junction. Interestingly, among the regulated pathways there are also the Signaling pathways regulating pluripotency of stem cells and cAMP signaling pathway that, together with the TGF-beta signaling pathway, have been observed to be regulated by targets of miRNAs detected in peripheral blood samples of AK and cSCC patients by Dańczak-Pazdrowska et al. [10]. On the other hand, Proteoglycans in cancer is the second most regulated pathway by cSCC miRNAs according to this article and by AK miRNAs according to our work, and other pathways have been commonly found, such as Pathways in cancer, Fatty acid biosynthesis, MAPK signaling pathway, and mTOR signaling pathway.

**Figure 2:**
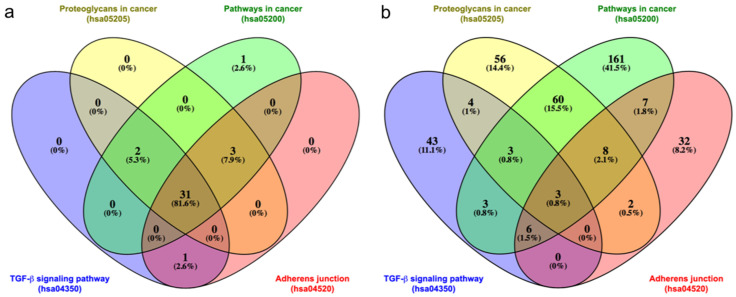
(**a**) Venn diagram representing the overlaps among the miRNAs regulating the first four KEGG pathways from Table 3. (**b**) Venn diagram representing the overlaps among all the target genes regulating the first four KEGG pathways from Table 3. Diagrams drawn using Venny 2.1 Internet site (https://bioinfogp.cnb.csic.es/tools/venny/, accessed on 23 May 2023). The list of the miRNAs and genes is reported in Supplementary Tables S1 and S2, respectively.

Given the elevated number of the AK modulated miRNAs involved in all the KEGG pathways, the significant overlap among miRNAs was confirmed by the Venn diagram, which showed that 31 miRNAs are commonly present in the four top regulated pathways (Figure 2a and Supplementary Table S1). Therefore, a Venn diagram was constructed to analyze the overlaps among the genes regulating TGF-beta signaling, Proteoglycans in cancer, Pathways in cancer, and Adherens junction. There is one high overlap of 60 genes among the miRNA targets controlling Proteoglycans in cancer and Pathways in cancer, but only 3 genes commonly regulate the top four pathways (Figure 2b and Supplementary Table S2).

The expression of some targets controlling Proteoglycans in cancer and Pathways in cancer, key factors in apoptosis and cell cycle, was analyzed by realtime RT-PCR: *TP53*, *MDM2*, *CDKN1A*, *CDK6*, and *CCND1*. Interestingly, p53 is a regulator of miR34a, whereas *MDM2* and *CDKN1A* are common targets of miR-19b, -146a, and -193b. *CDK6* is shared by miR-19b, -34a, -186, -193b, and -214, and *CCND1* is a target of miR-19b, -31, -34a, and -193b. Except for MDM2, we observed the modulation of the mRNAs of these genes in AK compared to HS samples (Table 4).

## 4. Discussion

A MiRNA profile of skin scraping samples was performed in RNA extracts obtained from pools of AK lesions and healthy-looking skin of the same individual. The results showed that 43 miRNAs are dysregulated in AK compared to HS samples, used as a control. The expression of seven miRNAs reported to play a role in skin cancer, namely miR-19b, -31, -34a, -126, -146a, -193b, and -222, was further analyzed by single qRT-PCR performed in the same AK and HS sample pools. Interestingly, a role in skin cancer has been reported for these miRNAs. MiR-19b belongs to the miR-17-92 cluster, known as oncomir since it is differentially expressed in different types of tumors, including cSCC [20]. MiR-31 and -222 are upregulated, whereas miR-34a is downregulated in cSCC [8,9,21]. MiR-19 and -126 seem to be early-stage specific markers of AK, while miR-193b is modulated throughout the malignant evolution of AK to cSCC [21]. MiR-146a is a modulator of inflammatory immune responses, and it has been reported to play a role in the development of both basal cell carcinoma and squamous cell carcinoma [22].

Among the other miRNAs differentially expressed in AK samples, miR-186, -203, and -214 also have been reported to be dysregulated in cSCC [23,24,25].

Our analysis with the DIANA tool mirPath v.3, performed with all the 43 miRNAs differentially expressed between the AK and HS samples, highlighted 66 pathways, most of which are regulated by targets of more than 30 miRNAs. Seventeen pathways are directly involved in cancer, because they are fundamental for the specific cancer progression or the principal pathways dysregulated in many cancers (i.e., Ras signaling, PI3K-Akt signaling, cAMP signaling), indicating their role in tumor development and confirming that AK could be a precursor of cSCC. In particular, the first two top regulated pathways are TGF-β signaling and Proteoglycans in cancer, followed by Pathways in cancer and Adherens junction. As expected, the miRNAs Venn diagram on these four pathways shows significant overlaps among miRNAs (31 common miRNAs), whereas the target genes Venn diagram highlights a significant overlap among the genes involved in the first two top pathways (60 genes) but only 3 genes commonly regulating all four pathways, i.e., SMAD2, RHOA, and MAPK1. The difference between the two Venn diagrams is due to the elevated number of target genes regulated by each miRNA.

TGF-β signaling pathway has a dual function, since its activation in healthy cells and in early-stage cancer induces cell-cycle arrest, whereas in late-stage cancer it can promote metastasis and chemoresistance. In this pathway, either SMAD or non-SMAD signaling can be activated. When activated, SMAD2 and SMAD3 are phosphorylated and form heterodimeric and trimeric complexes with SMAD4 that regulate the expression of target genes of the TGF-β signaling pathway [26]. As shown by the DIANA tool mirPath v.3 analysis, SMAD2 is a target of miR-186, SMAD3 of miR-214, and SMAD4 of both miR-34a and -146a. As reported by Cottonham et al. [27], TIAM1 (T lymphoma and metastasis gene 1) is a protein involved in the TGF-β signaling pathway, and it is a target of miR-31. Suppression of this protein leads to increased migration and invasion of a colon rectal cancer cell line, but its role in several types of cancer may be different. TIAM1 inhibits tumorigenesis in a Ras-induced skin cancer model, and miR-31 may be a negative regulator of metastasis development in breast cancer.

Our analysis highlighted that miR-19b, -146a, -186, -193b, and -203 share the target ErbB4, a member of the ErbB receptor family, also known as the EGF receptor family, which is involved in human cancer. ErbB receptors bind to many signaling proteins, inducing the activation of different signaling pathways, thus regulating several critical cellular processes, such as cell proliferation, differentiation, survival, metabolism, and migration. ErbB4 has both oncogenic and tumor suppressor functions even if in tumors it has been more frequently observed downregulated rather than upregulated [28]. ErbB4 is involved in the chondroitin sulfate (CS) and dermatan sulfate (DS) proteoglycan pathway. Both CS and DS regulate critical cellular processes, such as proliferation, apoptosis, migration, adhesion, and invasion. The CS/DS side chains of chondroitin sulfate proteoglycans take part in different interactions within the extracellular matrix and, therefore, have a key role in the regulation of proliferation, apoptosis, migration, adhesion, and invasion [29]. High levels of melanoma-associated chondroitin sulfate proteoglycans have been reported in melanoma, resulting in increased integrin function, activation of Erk1/2, cell growth, and motility [30]. ErbB2 is a well-established oncogene that is involved in different pathways in cancer [28], and the DIANA tool mirPath v.3 analysis showed that ErbB2 is a common target of miR-34a and -214, whereas miR-193b and -222 share the target PTEN, a known potent tumor suppressor, frequently mutated in human cancer [31].

During UV-induced DNA damage, the regulation of factors involved in apoptosis, cell growth, and cell cycle is pivotal to determine the fate of cells toward tumorigenesis. The DIANA tool mirPath v.3 analysis shows that in Pathways in cancer converge different pathways, among which are p53-signaling pathway and Cell cycle, regulated by common genes, such as *p53*, *MDM2*, *CDKN1A*, *CDK6*, and *CCND1*. Interestingly, these factors are targets of a group of miRNAs modulated in the AK samples. *MDM2* and *CDKN1A* are common targets of miR-19b, -146a, and -193b. *CDK6* is shared by miR-19b, -34a, -186, -193b, and -214. *CCND1* is a target of miR-19b, -31, -34a, and -193b. The tumor suppressor p53 is a regulator of miR34a that, in turn, regulates p53 through its target *SIRT1* [32]. Except for *MDM2*, the modulation of the mRNAs of p53, *CDKN1A*, *CDK6*, and *CCND1* we observed in the AK compared to HS samples.

It has been demonstrated that miR-34a regulates the activity of p53 in cells that undergo palmitate-induced lipoapoptosis [33]. After exposure to saturated fatty acids, such as palmitate acid, the expression of the transcription factor FOXO3 is upregulated, leading to an increment in miR-34a levels. This miRNA downregulates SIRT1, an enzyme that inactivates p53 via deacetylation, and cMET and KLF4, two anti-apoptotic factors. Consequently, the higher levels of active p53 could lead to apoptosis, thus blocking cancer progression [33]. Palmitate-induced apoptosis (PA) is a side process linked to the lipid biogenesis that can regulate miR-126 expression, whose target is the TNF receptor-associated factor, TRAF7. One of the main effects of PA on cells is the production of ROS and other factors that lead to apoptosis, namely through activation of the JNK and NF-κB pathways by TRAF7. MiR-126 blocks the translation of TRAF7 and prevents its recognition by TNF-α, but when PA is present, miR-126 is inhibited and the cell can undergo apoptosis [34]. In this study, we observed increased levels of miR-126 in AK-positive skin in comparison with healthy skin; therefore, it is possible to hypothesize that AK cells could regulate palmitic acid biosynthesis to prevent apoptosis and maintain tumor progression. Indeed, miR-126 is involved in fatty acid biosynthesis, regulating the expression of FASN enzymes such as ACSL1 and Insig1. In particular, it has been shown that in mammary luminal epithelial cells, inhibition of miR-126 leads to a decrease in both protein levels and in the number of cytoplasmic lipid droplets [35].

The biosynthesis of fatty acids may also be influenced by miR-222 and miR-31-3p [36], which share the target *ACOX1*, a catalytic rate-limiting gene in the β-oxidation of fatty acids from triglycerides, in particular miR-31 during oral squamous cell carcinoma (OSCC) and head and neck squamous cell carcinoma (HNSCC). Alterations in the lipidosome of OSCC caused by ACOX1 silencing enhance the motility and fitness of these tumor cells [37]. In our experiments, both miR-222 and miR-31 were upregulated in AK cells. Since AK could be considered a pre-malignant form of cSCC, it is possible to presume that the upregulation of these two miRNAs could indicate a transition from in situ carcinoma to full-blown epithelial cancer. Furthermore, considering that miR-34 and miR-126 are both involved in the silencing of genes that regulate apoptosis through fatty acid biosynthesis and catalysis [33,35], it is possible that the concertation between miR-222 and miR-31 to dysregulate palmitate and other free fatty acids could promote cancer progression.

Qian et al. [38] reported that exosomes from adipose mesenchymal cells are capable of paracrine gene regulation of cell renewal and proliferation during epithelial tissue healing. The mechanism behind this process has to do with the silencing of SOX9 by miR-19b. SOX9 is a crucial transcription factor that is involved in skin healing as it activates cell proliferation and differentiation through the Wnt/β-catenin pathway. The elevated levels of miR-19b that we observed in subjects with AK may be a physiological response to repair skin lesions inflicted by AK.

Epithelial cells exhibit several types of cell–cell junctions that play a key role in the maintenance of epithelial homeostasis. Dysregulation of molecules in the junctions promotes cell migration and tumor metastasis. In the adherens junctions, the cytoplasmic domain of E-cadherin forms a ternary complex with β-catenin and α-catenin, which in turn binds to F-actin, linking the catenin-based complexes to the actin cytoskeleton [39]. α-catenin (CTNNA)1, which is widely expressed in normal human tissues and in many malignancies, inhibits adhesion, invasion, and induces apoptosis of tumor cells by promoting or collaborating with E-cadherin. The expression of CTNNA1 is downregulated in different types of tumors, and it is often associated with reduced expression of other proteins of E-cadherin–catenin cell adhesion complex [40]. Among the top regulated pathways in our DIANA tool mirPath v.3 analysis is Adherens junction, where CTNNA1 and CTNNA2 are key factors. These two molecules are targets of miR-214 and -186, respectively, indicating that the upregulation of such miRNAs detected by the TaqMan Array can contribute to reduce the expression of catenin α and facilitate cell migration.

In skin cancer development, UV radiation is the main risk factor; however, virus infection, in particular human papillomavirus (HPV) infection, also seems to play a significant role. Progression of AK to cSCC is a multifactorial event, and many findings support the role of β-HPVs in the early steps of the carcinogenetic process, in cooperation with UV radiation [41]. We previously showed a large spectrum of β- and γ-HPVs in healthy-looking and lesion skin of patients affected by AK [11,12]. Interestingly, these samples belonged to the same cohort of patients as the present work; therefore, it is likely that in the AK and HS analyzed for miRNA expression, β- and γ-HPVs are abundantly present, contributing to modulate miRNA expression. Indeed, we showed that β-38 and -49 HPV E6 and E7 protein expression in primary human keratinocytes leads to the modulation of miRNAs involved in tumorigenesis [42,43].

## 5. Conclusions

Skin undergoes continuous renewal, and scrape cytology is a simple and cost-effective technique useful for the rapid diagnosis of some tumors [12]. In this study, scraping samples of actinic keratosis lesions proved to be useful in identifying different microRNAs that are modulated compared to healthy skin samples. However, further studies involving large numbers of samples are needed to confirm the hypothesis that the observed dysregulation of miRNAs in the AK samples may represent the combined result of UV exposure and human papillomavirus infection.

## Figures and Tables

**Table 1:** Primers used in Real Time RT-PCR.

Gene	FWD 5′–3′	REV 5′–3′	Ref.
*TP53*	CCTCAGCATCTTATCCGAGTGG	TGGATGGTGGTACAGTCAGAGC	[14]
*MDM2*	TGGGCAGCTTGAAGCAGTTG	CAGGCTGCCATGTGACCTAAGA	[15]
*CDKN1A*	GGCAGACCAGCATGACAGATT	GCGGATTAGGGCTTCCTCTT	[16]
*CDK6*	GCTGACCAGCAGTACGAATG	GCACACATCAAACAACCTGACC	[17]
*CCND1*	CCGTCCATGCGGAAGATC	GAAGACCTCCTCCTCGCACT	[18]

**Table 2:** List of dysregulated miRNAs in AK compared to HS, detected by TaqMan Array Human MicroRNA A Card analysis.

	MicroRNAs	Accession Number	Fold Change
1	hsa-miR-16-5p	MIMAT0000069	238.911
2	hsa-miR-150-5p	MIMAT0000451	177.837
3	hsa-miR-494-3p	MIMAT0002816	177.398
4	hsa-miR-31-5p	MIMAT0000089	177.057
5	hsa-miR-203a-5p	MIMAT0031890	160.851
6	hsa-miR-191-5p	MIMAT0000440	130.259
7	hsa-let-7e-5p	MIMAT0000066	110.853
8	hsa-miR-222-3p	MIMAT0000279	99.651
9	hsa-miR-146a-5p	MIMAT0000449	85.494
10	hsa-miR-19b-3p	MIMAT0000074	75.58
11	hsa-miR-200c-3p	MIMAT0000617	58.549
12	hsa-miR-320a	MIMAT0000510	56.054
13	hsa-let-7b-5p	MIMAT0000063	51.857
14	hsa-miR-24-3p	MIMAT0000080	48.505
15	hsa-miR-374-5p	MIMAT0000727	48.309
16	hsa-miR-126-3p	MIMAT0000445	47.816
17	hsa-miR-422a	MIMAT0001339	44.507
18	hsa-miR-146b-3p	MIMAT0004766	43.107
19	hsa-miR-486-5p	MIMAT0002177	40.989
20	hsa-miR-193b-3p	MIMAT0002819	40.612
21	hsa-miR-454-3p	MIMAT0003885	34.13
22	hsa-miR-484	MIMAT0002174	24.427
23	hsa-miR-186-5p	MIMAT0000456	23.968
24	hsa-miR-504-5p	MIMAT0002875	20.504
25	hsa-miR-342-3p	MIMAT0000753	19.67
26	hsa-miR-487a-3p	MIMAT0002178	16.643
27	hsa-miR-195-5p	MIMAT0000461	16.126
28	hsa-miR-518f-3p	MIMAT0002842	15.495
29	hsa-miR-200a-3p	MIMAT0000682	15.161
30	hsa-miR-636	MIMAT0003306	13.205
31	hsa-miR-618	MIMAT0003287	12.183
32	hsa-miR-218-5p	MIMAT0000275	11.297
33	hsa-miR-302c-3p	MIMAT0000717	7.095
34	hsa-miR-214-3p	MIMAT0000271	3.675
35	hsa-miR-145-5p	MIMAT0000437	3.417
36	hsa-miR-519e-3p	MIMAT0002829	1.342
37	hsa-miR-182-5p	MIMAT0000259	1.296
38	hsa-miR-211-5p	MIMAT0000268	1.143
39	hsa-miR-518b	MIMAT0002844	1.023
40	hsa-miR-208b-3p	MIMAT0004960	0.272
41	hsa-miR-34a-5p	MIMAT0000255	0.173
42	hsa-miR-127-5p	MIMAT0004604	0.108
43	hsa-miR-370-3p	MIMAT0000722	0.083

Fold change: 2^−ΔΔCT^.

**Table 3:** MiRNA pathway analysis by DIANA mirPath v.3 on KEGG pathways linked to the 43 selected miRNAs.

	KEGG Pathway	*p*-Value	# Genes *	# miRNAs **
1.	TGF-beta signaling pathway (hsa04350)	7.30 × 10^−10^	62	34
2.	Proteoglycans in cancer (hsa05205)	7.30 × 10^−10^	136	36
3.	Pathways in cancer (hsa05200)	3.36 × 10^−9^	251	37
4.	Adherens junction (hsa04520)	1.84 × 10^−8^	58	35
5.	Axon guidance (hsa04360)	1.84 × 10^−8^	91	37
6.	Hippo signaling pathway (hsa04390)	2.70 × 10^−7^	99	37
7.	Fatty acid biosynthesis (hsa00061)	1.62 × 10^−6^	7	12
8.	Ras signaling pathway (hsa04014)	4.68 × 10^−6^	142	36
9.	ErbB signaling pathway (hsa04012)	1.32 × 10^−5^	61	35
10.	Glioma (hsa05214)	1.65 × 10^−5^	46	34
11.	Wnt signaling pathway (hsa04310)	1.78 × 10^−5^	95	35
12.	Melanoma (hsa05218)	3.69 × 10^−5^	53	32
13.	Signaling pathways regulating pluripotency of stem cells (hsa04550)	3.69 × 10^−5^	94	37
14.	Rap1 signaling pathway (hsa04015)	5.55 × 10^−5^	134	35
15.	GABAergic synapse (hsa04727)	0.0001604280	54	35
16.	Thyroid hormone signaling pathway (hsa04919)	0.0001726085	77	36
17.	Estrogen signaling pathway (hsa04915)	0.0002032496	62	34
18.	MAPK signaling pathway (hsa04010)	0.0002242160	160	37
19.	Renal cell carcinoma (hsa05211)	0.0002295720	48	33
20.	Glycosaminoglycan biosynthesis—heparan sulfate/heparin (hsa00534)	0.0005046348	18	21
21.	Choline metabolism in cancer (hsa05231)	0.0005046348	69	34
22.	Neurotrophin signaling pathway (hsa04722)	0.0005046348	81	37
23.	Focal adhesion (hsa04510)	0.0007711823	129	36
24.	Glycosphingolipid biosynthesis—lacto and neolacto series (hsa00601)	0.0009080097	17	19
25.	FoxO signaling pathway (hsa04068)	0.0009080097	85	35
26.	Prostate cancer (hsa05215)	0.0010195428	60	33
27.	Glutamatergic synapse (hsa04724)	0.0020494968	71	34
28.	PI3K-Akt signaling pathway (hsa04151)	0.0020578098	199	37
29.	Regulation of actin cytoskeleton (hsa04810)	0.0032897905	131	35
30.	Amphetamine addiction (hsa05031)	0.0033806984	43	28
31.	Thyroid hormone synthesis (hsa04918)	0.0040209555	44	31
32.	Colorectal cancer (hsa05210)	0.0040209555	42	33
33.	Oocyte meiosis (hsa04114)	0.0040209555	72	35
34.	Prolactin signaling pathway (hsa04917)	0.0043891560	47	33
35.	Insulin secretion (hsa04911)	0.0052671844	56	35
36.	cAMP signaling pathway (hsa04024)	0.0059360368	120	35
37.	Chronic myeloid leukemia (hsa05220)	0.0060741359	48	35
38.	Pancreatic cancer (hsa05212)	0.0066389106	45	32
39.	Thyroid cancer (hsa05216)	0.0068643563	22	28
40.	Endometrial cancer (hsa05213)	0.0078889298	35	31
41.	Phosphatidylinositol signaling system (hsa04070)	0.0078889298	52	34
42.	Adrenergic signaling in cardiomyocytes (hsa04261)	0.0078889298	89	34
43.	Vasopressin-regulated water reabsorption (hsa04962)	0.0079518053	31	26
44.	Oxytocin signaling pathway (hsa04921)	0.0081386021	99	35
45.	Non-small-cell lung cancer (hsa05223)	0.0082341417	37	32
46.	cGMP-PKG signaling pathway (hsa04022)	0.0082341417	100	36
47.	Transcriptional misregulation in cancer (hsa05202)	0.0083247611	104	38
48.	Long-term potentiation (hsa04720)	0.0097964993	45	32
49.	mTOR signaling pathway (hsa04150)	0.0104774872	42	32
50.	Endocytosis (hsa04144)	0.0105456910	121	36
51.	Long-term depression (hsa04730)	0.011338320	39	32
52.	Viral carcinogenesis (hsa05203)	0.011338320	102	36
53.	Vascular smooth muscle contraction (hsa04270)	0.011568669	69	34
54.	Gap junction (hsa04540)	0.015258034	51	35
55.	Ubiquitin mediated proteolysis (hsa04120)	0.021158638	81	37
56.	Hepatitis B (hsa05161)	0.021158638	84	37
57.	Biotin metabolism (hsa00780)	0.022422627	2	4
58.	Morphine addiction (hsa05032)	0.027484035	53	36
59.	Inflammatory mediator regulation of TRP channels (hsa04750)	0.028782594	60	31
60.	N-Glycan biosynthesis (hsa00510)	0.032645322	27	24
61.	Nicotine addiction (hsa05033)	0.034469917	26	28
62.	Hedgehog signaling pathway (hsa04340)	0.035921144	34	24
63.	Gastric acid secretion (hsa04971)	0.036593705	48	34
64.	Melanogenesis (hsa04916)	0.038811067	63	33
65.	Tight junction (hsa04530)	0.038811067	81	37
66.	Bacterial invasion of epithelial cells (hsa05100)	0.043438632	47	32

# represents the number of genes affected * and of miRNAs involved ** for each pathway.

**Table 4:** Gene expression analysis performed in AK compared to HS. Total RNA from each sample was purified as described in Materials and Methods. Results were expressed as fold of induction using the 2^−ΔΔCT^ method using HPRT-1 as calibrator and HS as control.

Gene	Fold Change	SD	*p* Value
*TP53*	39.946	3.603	<0.001
*MDM2*	0.898	0.234	0.224
*CDKN1A*	161.751	0.518	<0.001
*CDK6*	3.371	1.417	<0.001
*CCND1*	204.732	33.863	<0.001

The reported values represent the mean of three independent experiments ± the SD. Fold change: 2^−ΔΔCT^.

## Data Availability

The row data are available upon specific request.

## Supplementary Materials

The following are available online at https://www.mdpi.com/article/10.3390/biomedicines11061719/s1, Table S1: MiRNAs in Venn diagram; Table S2: Target genes in Venn diagram.
